# Phosphorus–Silicon Additive Increases the Mechanical and Fire Resistance of Epoxy Resins

**DOI:** 10.3390/ma18122753

**Published:** 2025-06-12

**Authors:** Zhe Wang, Shuaijun Guo, Wenwen Yu, Xiaohong Liang

**Affiliations:** College of Materials Science & Engineering, Taiyuan University of Technology, Taiyuan 030024, China; wangzhe0243@link.tyut.edu.cn (Z.W.); 2023310047@link.tyut.edu.cn (S.G.); yuwenwen@tyut.edu.cn (W.Y.)

**Keywords:** LOI test, cone calorimetry, resin matrix, high-performance epoxy resins, flame-retardant P-Si

## Abstract

Epoxy resins are limited by their flammability and brittleness. In this study, a phosphorus- and silicon-based additive was synthesized to improve fire resistance and mechanical performance. The incorporation of just 1 wt% phosphorus from this additive into epoxy resin achieved a limiting oxygen index of 33% and a V-0 fire rating. The modified epoxy exhibited a 52.43% reduction in the peak heat release rate and a 35.70% decrease in total smoke production compared to the unmodified resin, demonstrating enhanced heat resistance and smoke suppression. Notably, the modified epoxy thermoset displayed superior mechanical properties, with tensile and impact strengths increasing by 48.41% and 130%, respectively. This research presents a promising approach for developing high-performance epoxy resins with improved flame retardancy, smoke suppression, and mechanical strength.

## 1. Introduction

Due to its exceptional adhesion, superior electrical insulation, and chemical stability, epoxy resin (EP) has found extensive applications in surface coatings, adhesives, and electronic product packaging [1,2,3]. However, cured EP exhibits some degree of flammability and brittleness, which restricts its further utilization in various industrial sectors. These limitations not only hinder its industrial applications but also pose risks to human lives, properties, and environmental safety [4,5,6,7]. Therefore, epoxy composite materials with excellent flame retardancy and good mechanical properties show an extremely broad development prospect.

In order to enhance the flame-retardant properties of epoxy resin (EP), researchers have shifted their focus towards halogen-free flame retardants such as phosphorus, silicon, nitrogen, and boron, which are considered safe for both human health and the environment [8,9,10,11,12]. Among these, phosphorus-based flame retardants like 9,10-dihydro-9-oxa-10-phosphaphenanthrene-10-oxide (DOPO) have emerged as promising candidates due to their high efficiency in flame retardancy, ability to promote char formation, and synergistic effects with other elements, making them compatible with a wide range of polymers [13,14,15]. During combustion, DOPO can generate PO· radicals that combine with active groups, thereby reducing the reactivity of the system [16]. Moreover, in the condensed phase, DOPO contributes to the formation of a dense char layer by producing phosphoric acid and other compounds, which helps in inhibiting the spread of flames [17]. Zhang et al. [18] have experimented with incorporating three phosphorus-containing flame retardants—DOPO, ammonium polyphosphate (APP), and pentaerythritol phosphate (PEPA)—into EP systems. When these flame retardants containing 0.9 wt% of phosphorus were added to EP, all the modified materials achieved a V-0 rating in vertical combustion tests. Notably, EP/DOPO samples exhibited higher limiting oxygen index (LOI) values of 32% and a lower peak heat release rate (pHRR) of 20.4%, highlighting the effectiveness of DOPO as a flame retardant. In another study by Zhang et al. [19], a reactive flame retardant derived from DOPO, known as PBI, was developed and incorporated into EP composites at a concentration of 10.8 wt%. The resulting material displayed an LOI of 34.6% and met the UL-94 V-0 standard, demonstrating the excellent flame-retardant properties of PBI.

While phosphorus-based flame retardants have been proven effective in enhancing flame retardancy, they often have a negative impact on the mechanical properties of materials, presenting a significant challenge [20,21]. Guo and colleagues [22] developed a flame retardant containing DOPO for epoxy resins, which resulted in a 6.5% increase in the limiting oxygen index (LOI) value. However, this improvement came at the cost of a 14% decrease in tensile strength, highlighting the need for a balanced approach to flame retardancy. Recent research has shown that synergistic systems combining phosphorus and silicon components hold promise in overcoming this trade-off [17,23]. Zhang et al. [24,25] observed a unique “blowing-out extinguishing effect” in an epoxy system modified with DOPO-POSS. This synergistic effect not only improves flame retardancy but also boosts overall performance, demonstrating a synergistic effect where the whole is greater than the sum of its parts. In a similar vein, Yu et al. [26] developed a multifunctional EP additive (ETP) by incorporating phosphorus-containing compounds into an organic silicone epoxy resin. The material achieved a remarkable LOI value of 29.8% during fire exposure with the addition of only 6 wt% ETP, along with a 114.5% increase in impact strength. This innovative approach showcases the potential of combining different flame-retardant components to achieve superior fire safety and mechanical performance.

The combined effects of phosphorus and silicon work together to improve flame retardancy and address the mechanical issues often associated with phosphorus additives [27,28]. Phosphorus–silicon flame retardants play a crucial role in enhancing the thermal stability and compactness of the char layer by promoting char formation during combustion, while simultaneously strengthening the interfacial bonding between EP and fillers to facilitate a more tightly interconnected three-dimensional network stricture and improve mechanical properties [29,30,31,32]. Gan et al. [33] developed a flame retardant, TMDS-DOPO, by combining flexible siloxane and DOPO, which, when incorporated at 4.20 wt%, improved the flexural strength and modulus of EP by 13% and 23% respectively, achieving a V-0 grade in vertical combustion tests. Zhang et al. [34] created a hyperbranched flame retardant, HPNSi, with a non-aromatic structure. When incorporated into EP, the impact strength and flexural strength of the HPNSi/EP material increased by 41% and 23% respectively, thanks to the unique branched structure and flexible Si-O segments of HPNSi. The phosphorus–silicon system has shown great promise in enhancing both the flame-retardant properties and mechanical strength of EPs simultaneously.

By leveraging the synergistic effect of phosphorus and silicon elements during flame retardancy, while simultaneously enhancing compatibility with the matrix material, a highly efficient flame-retardant system can be formed. In this study, a phosphorus- and silicon-containing flame retardant was prepared using DOPO and 1,3-tetramethyldivinyldisiloxane and applied to the modification of EP. A comprehensive analysis of the material was conducted using standard flame retardancy tests (LOI test, vertical burning test, and cone calorimeter test), mechanical property evaluations (DMA test, tensile impact test), and detailed flame retardancy mechanism studies (TG-TR, XPS, Raman spectroscopy). This study provides a comprehensive characterization of the modified EP material, improves its mechanical properties, proposes a novel synthesis method, and ultimately expands the practical applications of EP.

## 2. Materials and Methods

### 2.1. Materials

Bisphenol A epoxy resin (EP, 99%) and azobisisobutyronitrile (AIBN, 98%+) were purchased from Macklin Biochemical Technology Co., Ltd. (Shanghai, China). 1,3-Tetramethyldivinyldisiloxane (TMVDCS, 98%+) was obtained from Aladdin Biochemical Technology Co., Ltd. (Shanghai, China). 9,10-Dihydro-9-oxa-10-phosphaphenanthrene-10-oxide (DOPO, 99%) was supplied by AiChun Biotechnology Co., Ltd. (Shanghai, China). 4,4′-Diaminodiphenylmethane (DDM, 97%) was provided by Merida Technology Co., Ltd. (Beijing, China). Toluene (99.5%) was acquired from Weihua Trading Co., Ltd. (Taiyuan, China).

### 2.2. Synthesis of the Flame Retardant

**Scheme 1:**
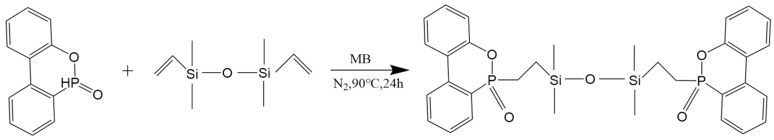
Synthetic route of P–Si flame retardant.

In this study, DOPO (0.1 mol, 21.6 g) and TMVDCS (0.05 mol, 9.32 g), along with 50 mL of toluene, were sequentially added to a 250 mL three-neck flask equipped with a reflux condenser. The mixture was heated to 90 °C under a nitrogen atmosphere with magnetic stirring. Following the complete dissolution of DOPO, AIBN (0.1 g) predissolved in toluene (30 mL) was slowly added dropwise into the reaction vessel. The reaction was maintained at this temperature for 24 h, followed by purification through filtration. After removing toluene using a rotary evaporator, a colorless liquid product was obtained. The synthesis route is illustrated in Scheme 1.

### 2.3. Incorporation into Epoxy Resin

The sample formulations of EP/P-Si flame-retardant composites are presented in Table 1. First, we mixed the EP with different proportions of P–Si group-containing flame retardants. This mixture was then heated to 90 °C with magnetic stirring, and subsequently stirred thoroughly for 1 h. Next, DDM was added to this mixture according to the ratio of epoxide and amino equivalent of 3:1, and then stirred thoroughly for 10 min. The mixture was poured into a mold and cured at 120 °C for 2 h, and then at 150 °C for 3 h to obtain the epoxy composite sample.

### 2.4. Spectroscopic Characterization

The synthesized P-Si flame retardant was characterized using spectroscopic techniques. FTIR spectra were obtained with an INVENIO-S FTIR spectrometer (Bruker, Karlsruhe, Germany) over the wavenumber range of 4000–450 cm^−1^. Nuclear magnetic resonance (NMR) spectroscopy was performed on a Bruker 400 MHz spectrometer (Bruker, Karlsruhe, Germany).

### 2.5. Thermal and Fire Resistance Tests

Differential scanning calorimetry (DSC) was measured with 204F-1 (Netzscg, Selb, Germany) under a nitrogen atmosphere at heating rates of 10 °C/min.

Thermogravimetric analysis (TGA) was performed using a TGA2 analyzer (Mettler Toledo, Greifensee, Switzerland) at a heating rate of 10 °C/min from 30 to 700 °C.

The limiting oxygen index (LOI) was determined using a PX-01-005 oxygen index meter (Fairman, Jiangsu, China) on specimens with dimensions of 130.0 mm × 6.5 mm × 3.0 mm, following the GB/T 2406-2009 standard [20]. Vertical combustion tests (UL-94) were conducted on splines (130.0 mm × 13.0 mm × 3.0 mm) using CZF-2 horizontal and vertical combustion testers (Fairman, Jiangsu, China) according to the GB/T 2408-2008 standard [20]. Cone calorimeter tests were performed on samples with dimensions of 100.0 mm × 100.0 mm × 3.0 mm using a conical calorimeter (VOUCH 6810, VOUCH, Suzhou, China) according to ISO 5660 [20].

### 2.6. Analysis of Flame Retardancy Mechanism

Thermogravimetric and Fourier transform infrared spectroscopy (TG-FTIR) was carried out by STA-2500 (Netzsch, Germany) and Fisher IS-50 (Thermo Fisher, Waltham, MA, USA). The sample was heated at a rate of 10 °C/min under a nitrogen flow of 50 mL/min in a temperature range from 30 to 800 °C.

X-ray photoelectron spectroscopy (XPS) measurements were performed using an electron spectrometer (K-Alpha, Thermo Fisher, Waltham, MA, USA) to investigate the coke residue’s composition.

Residual char samples were analyzed by Raman spectroscopy (Horiba, Kyoto, Japan) for graphitization degree assessment.

### 2.7. Mechanical Property Tests

Standard rectangular specimens (60.0 mm × 10.0 mm × 4.0 mm) of different EP materials were prepared. Dynamic mechanical analysis was performed using a DMA 242 E instrument (Netzsch, Germany) in a dynamic single-cantilever mode at a heating rate of 3 °C/min, over a temperature range of 30 to 230 °C.

Dumbbell-shaped specimens (80.0 mm × 10.0 mm × 4.0 mm) were prepared for tensile testing, which was conducted using an Instron 6800 testing machine (Instron, Norwood, MA, USA) at room temperature, with a tensile speed of 2 mm/min. Standard rectangular specimens (60.0 mm × 10.0 mm × 4.0 mm) were prepared for notched impact testing using an Instron CEAST 9050 pendulum impact tester (Instron, Norwood, MA, USA) at room temperature. The fracture surface morphology of samples from the notched impact tests was examined using a Gemini SEM 360 field emission scanning electron microscope (Zeiss, Oberkochen, Germany).

### 2.8. Transparency Test

Light transmittance values of samples of 35 mm× 35 mm × 1 mm were determined in the range of 250–800 nm using an ultraviolet-visible (UV-vis) spectrophotometer (PE LAMBDA 1050, PerkinElmer, Shelton, CT, USA).

## 3. Results and Discussion

### 3.1. Structural Characterization of P–Si

**Figure 1:**
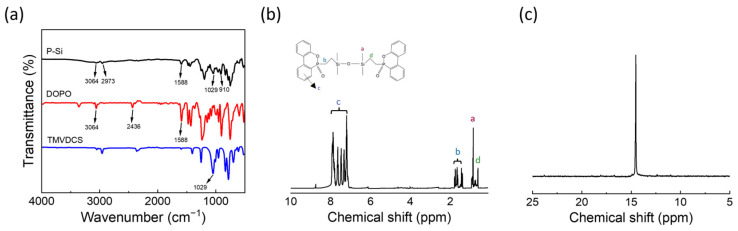
(**a**) IR spectra of DOPO, TMVDCS, and P–Si, (**b**) ^1^H NMR spectra, and (**c**) ^31^P NMR spectra.

Figure 1a depicts the IR spectra of DOPO, TMVDCS, and P–Si. In the spectrum of DOPO, the peak at 2436 cm^−1^ corresponded to the stretching vibration of P-H. In the spectrum of P–Si flame retardant, the peak at 2436 cm^−1^ completely disappeared, which indicate that DOPO had fully been reacted. The peak at 2973 cm^−1^ was attributed to the stretching vibration of the C-H bond connected to Si. The peaks at 3064 cm^−1^ and 1588 cm^−1^ were characteristic absorption peaks of the benzene ring. The stretching vibration peak of Si-O appeared at 1029 cm^−1^, which could also be observed in the TMVDCS spectrum, confirming the presence of a Si-O main chain in the synthesized P–Si structure. Both DOPO and P-Si spectra showed an absorption peak at 910 cm^−1^, which was attributed to the stretching vibration of P-O. This result indicates that the P-O bond had been introduced into the P–Si flame retardant. In the ^1^H NMR spectrum (Figure 1b), the peak around 0.7 ppm corresponds to Si-CH_3_, the chemical shift at 0.86 ppm corresponds to Si-CH_2_, the peaks in the range of 1.2–1.3 ppm correspond to P-CH_2_, and the peaks in the range of 7.2–7.9 ppm correspond to the hydrogen atoms on the benzene ring of P-Si. In the ^31^P NMR spectrum of P-Si (Figure 1c), only a single chemical shift peak was observed at 14.53 ppm, indicating that the synthesized P-Si contained phosphorus elements in only one chemical environment.

### 3.2. Curing Behavior

**Figure 2:**
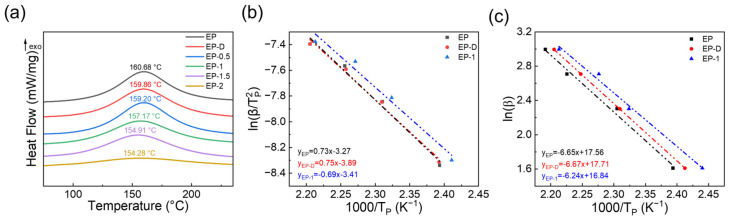
(**a**) DSC curves; fitting curves for (**b**) Kissinger and (**c**) Ozawa methods.

The non-isothermal DSC method was performed to evaluate the curing effect of P-Si towards EP. Figure 2 and Table 2 show the DSC curves and curing characteristics. Figure 2a exhibits a single exothermic peak, indicating a one-stage curing reaction. Compared to the neat EP, the EP/P-Si composite demonstrated a lower Tonset, TP, and Tend, suggesting that the P-Si flame retardant acts as a catalyst for the epoxy curing process [35]. Furthermore, the ΔH ∞ of the EP/P-Si system shifted to higher values, demonstrating an enhanced degree of curing completion in the composite system [36].

The Curing Index (CI) was determined to assess the curing process of EP using Equations (1)–(3) [22,37]. This index serves to indicate the degree of curing. Based on the calculated CI, the curing state of the EP/P-Si composite was deemed to be excellent. These findings suggest that the curing reaction of the EP, when modified by P-Si, was significantly enhanced.

(1) CI=∆T*×∆H*

(2) $$∆T*=\frac{∆T_{C}}{∆T_{Ref}}$$

(3) $$∆H*=\frac{∆H_{C}}{∆H_{Ref}}$$

Δ*T_C_*: Δ*T* for the EP/P-Si system, Δ*T_Ref_*: Δ*T* for the EP system, Δ*H_C_*: Δ*H* for the EP/P-Si system, and Δ*H_Ref_*: Δ*H* for the EP system.

In addition, the determination of the activation energies for various EP systems during the curing process can be achieved using the approaches developed by Kissinger and Ozawa (Equations (4) and (5)) [38,39].

(4) $$\ln\left(\frac{\beta }{T_{p}^{2}}\right)=\ln\left(\frac{AR}{E_{a}}\right)-\frac{E_{a}}{RT_{p}}$$

(5) $$\ln\beta =\ln\left(\frac{AE_{a}}{R}\right)-\frac{{1.052E}_{a}}{RT_{p}}-5.331$$

where *β* is the heating rate, *T_P_* is the exothermic peak temperature, *A* is the pre-exponential factor, and R is the ideal gas constant (8.314 J/mol·K).

The graphs in Figure 2b,c display the linear correlation of ln(β/$T_{P}^{2}$) and lnβ with 1/T_P_ × 10^3^, illustrating the applicability of both the Kissinger and Ozawa methods in this particular experimental setup. Table S1 provides a summary of the apparent activation energy (*Ea*) values derived from the fitted curves. The inclusion of P-Si resulted in a notable decrease in the activation energy of the epoxy curing process, thanks to the enhanced compatibility brought about by the P-Si flame retardant. Additionally, the weak adsorption interactions between phosphorus and silicon served as a catalyst for the curing reaction within the matrix.

### 3.3. Thermal Stability

**Figure 3:**
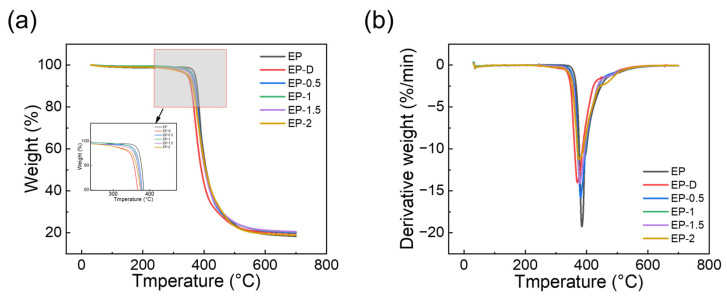
(**a**) TG and (**b**) DTG curves of the epoxy composites.

The thermal stability of the epoxy composites was evaluated using TG analysis. Figure 3 displays the TG and DTG curves of each sample under a nitrogen atmosphere, with detailed data presented in Table 3. All samples showed a single peak, indicating a single decomposition process at a temperature range of 290–490 °C (Figure 3a). The T_5%_ value of the EP/P-Si composites was significantly higher than the curing temperature of pure EP (150 °C), suggesting that the flame-retardant P-Si remained stable during the curing process and was effective as a flame retardant for EPs. The incorporation of the flame-retardant P-Si reduced the T_5%_ and T_Max_ of the EP composite, which is attributed to the earlier decomposition of P-Si compared to pure EP [40]. Moreover, at 700 °C, the carbon residue increased from 18.39% for EP to 21.58% for EP-1, demonstrating the epoxy composite’s improved coking ability with the addition of P-Si flame retardant. Remarkably, the CY700 values of the EP/P-Si composites were higher than those of the epoxy material with DOPO alone, highlighting a significant enhancement in thermal stability. This enhancement was attributed to the synergistic effect of phosphorus and silicon in promoting the formation of a thermally stable char layer [12,33]. A higher carbon residue suggests that the matrix materials generate less heat and fewer combustibles during combustion, enhancing the flame retardancy of the epoxy composite [41,42]. These findings indicate that the flame-retardant P-Si-modified EP generated more residual carbon during combustion, forming a carbon layer to inhibit further burning and reduce the burning rate.

### 3.4. Flame-Retardant Properties

The combustion properties of the epoxy composites were assessed using the Cone Calorimetry Test (CCT), UL-94, and limiting oxygen index (LOI) tests. Table 4 presents the vertical combustion rating test data and LOI values for the epoxy composites, while Figure S1 illustrates the state of the spline vertical combustion. During the combustion test, the pure EP sample produced thick smoke upon ignition, and continuous black drips ignited the standard cotton pad. The UL-94-grade test was unsuccessful, with an LOI value of only 23%. In contrast, the EP-0.5 sample exhibited an increased LOI value of 29% and did not produce any drips during combustion. The EP-0.5 sample showed a self-extinguishing capability after a brief period of combustion, achieving a UL-94 V-1 grade. In the case of the EP-1 sample, the spline quickly extinguished upon removal from the fire. It achieved a UL-94 V-0 grade and a 33% LOI value, indicating a significant enhancement in the flame retardancy of the EP. This improvement in flame retardancy performance was attributed to the synergistic effect of the various elements present in the P–Si structure. The phosphorus components underwent pyrolysis to generate phosphoric acid derivatives, which reacted with the -OH groups in the epoxy matrix through esterification and dehydration. This promoted the carbonization of EP to form a dense protective layer. Simultaneously, the silicon elements in the flame retardant created Si-C and SiO2 structures with the matrix during combustion, aiding in the formation of a stable carbon layer that could interrupt the combustion reaction and release noncombustible gas [43,44,45]. However, it was observed that the LOI value started to decline when the phosphorus content reached 1.5%. This was due to the excessively high P content hindering the dispersion of the flame retardant in the resin matrix, thereby reducing its effectiveness in enhancing flame retardancy. A comparison of the flame retardancy between this study and relevant literature from the past five years is detailed in Table S2. Sample EP-1 demonstrated an exceptional performance, achieving an LOI of 33% and a UL-94 V-0 rating.

**Figure 4:**
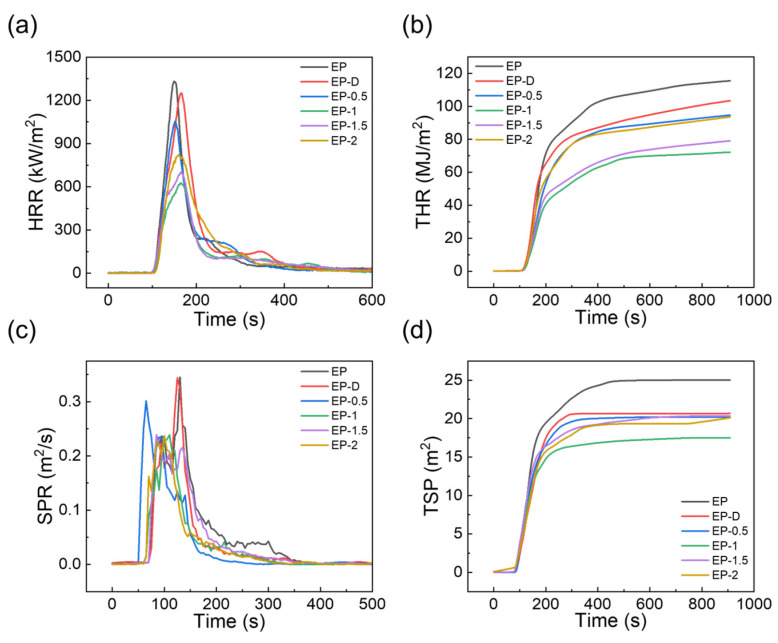
(**a**) HRR, (**b**) THR, (**c**) SPR, and (**d**) TSP curves.

In order to thoroughly assess the fire risk associated with EP composites, CCTs were carried out to provide a comprehensive evaluation of the flame retardancy of the composites. The results of the tests are presented in Figure 4 and summarized in Table 5. The EP/P–Si composites had a higher TTI than the pure EP, indicating that the addition of the flame retardant made the epoxy more resistant to ignition. Figure 4a demonstrates that the THR and pHRR of the flame-retardant materials were lower than those of the pure EP. Specifically, the EP-1 composite exhibited THR and pHRR values of 74.72 MJ/m^2^ and 629.11 kW/m^2^, respectively, which were 35.70% and 52.43% lower than those of the pure EP (116.2 MJ/m^2^ and 1322.55 kW/m^2^, respectively). These findings confirm that the inclusion of the P–Si flame retardant significantly delayed the HRR of the EP during combustion, thereby enhancing its flame retardancy. The emission of smoke and toxic gases during combustion was also analyzed, with the results depicted in Figure 4c,d and detailed in Table 5. It was observed that the addition of the P–Si flame retardant to the epoxy materials led to a significant decrease in the SPR, TSP, av-COY, and av-CO_2_Y. Specifically, the TSP value of the pure EP decreased from 25.01 m^2^ to 17.49 m^2^ for EP-1, representing a reduction of 30.07%. This reduction in smoke production indicates an improvement in the fire safety properties of the EP composites when treated with the P–Si flame retardant.

The flame retardancy mode was clarified via calculating the amounts of three different effects according to Equations (6)–(8) [46,47,48].

(6) $$Flame\;inhibition\;effect=1-\frac{{EHC}_{FREP}}{{EHC}_{EP}}$$

(7) $$Charing\;effect=1-\frac{{TML}_{FREP}}{{TML}_{EP}}$$

(8) $$Barrier\;and\;protective\;effect=1-\frac{\frac{{pHRR}_{FREP}}{{pHRR}_{EP}}}{\frac{{THR}_{FREP}}{{THR}_{EP}}}$$

where FREP and EP are flame-retardant epoxy resin and epoxy resin, respectively.

Upon comparing the three values presented in Table S3, it is evident that the EP/P-Si material showcases superior performance in terms of flame retardancy. In direct comparison to EP-D, the EP/P-Si material displays a higher level of charring. Additionally, the av-EHC for samples containing P-Si was 17.0 MJ/kg lower than that of EP alone. This solidifies the notion that P-Si effectively enhances flame retardancy by promoting the formation of a char layer, which serves to insulate the substrate thermally and inhibit the release of flammable gases.

The extensive findings from these studies offer strong evidence that the P-Si flame-retardant system significantly boosts fire resistance and successfully reduces the amount of smoke and toxic gases produced when materials combust.

### 3.5. Gas Phase Analysis

**Figure 5:**
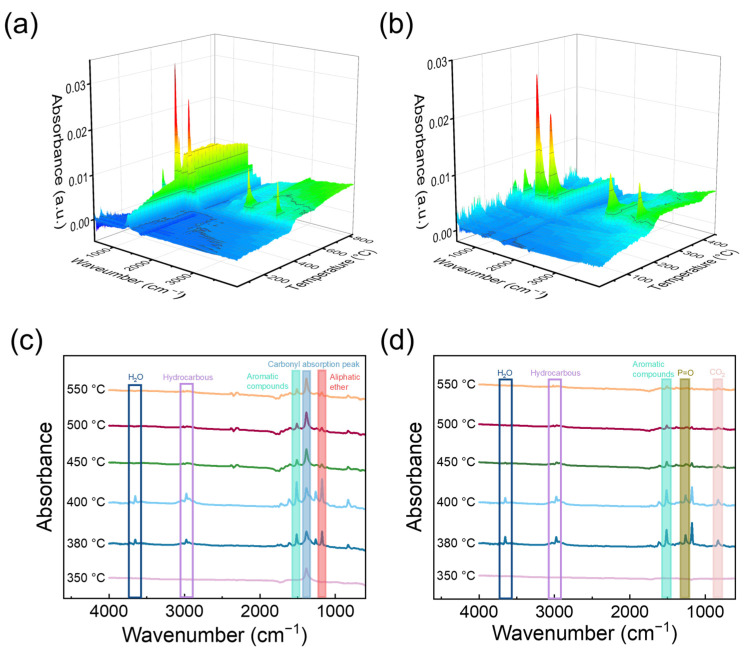
Three-dimensional TG-FTIR data of (**a**) EP and (**b**) EP-1. Characteristic spectra of (**c**) EP and (**d**) EP-1 pyrolysis products at different temperatures.

Through the implementation of thermogravimetric–infrared (TG-IR) coupling, we were able to gain into the changes in the gaseous products that result from the thermal decomposition of EP/P–Si composite materials. An analysis of the infrared 3D spectra (as depicted in Figure 5a,b) revealed the peak intensity of gaseous products for EP-1 (approximately 0.025) exhibited a noteworthy decrease of 16.17% in comparison to that of pure epoxy material (approximately 0.03). Furthermore, the maximum absorption intensities associated with decomposition products such as hydrocarbons (2990 cm^−1^), carbon monoxide (1620 cm^−1^), and aromatic compounds (1510 cm^−1^) had all been notably diminished. This signified that the incorporation of the flam- retardant P–Si has effectively curtailed the production of pyrolysis products from the epoxy resin composites.

Figure 5c,d shows the characteristic IR spectra of EP and EP-1 pyrolysis products at different temperatures. No significant signals were detected for EP-1 before reaching 380 °C (Figure 5d). The result indicates that the addition of P–Si can improve the thermal stability of EP. It can be observed from Figure 5d that a new characteristic absorption peak of P=O appeared at 1278 cm^−1^. The products resulted from the decomposition of the flame-retardant P–Si, with the phosphorus component being released into the gas phase, playing a role in inhibiting the combustion process. P-Si effectively suppressed the fuel supply by reducing the generation of combustible pyrolysis products. In particular, the peaks corresponding to smoke precursors (at 1510 cm^−1^, 830 cm^−1^) were significantly diminished [49].

### 3.6. Char Morphology and Chemical Analysis

**Figure 6:**
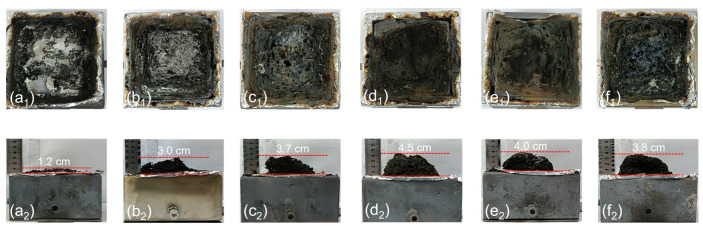
Digital photograph of the char residue. (**a_1_**_,_**a_2_**) EP, (**b_1_**,**b_2_**) EP-D, (**c_1_**,**c_2_**) EP-0.5, (**d_1_**,**d_2_**) EP-1, (**e_1_**,**e_2_**) EP-1.5, and (**f_1_**,**f_2_**) EP-2.

Figure 6 shows digital photographs of the coke residues after cone calorimeter testing. The top-view images revealed striking differences in char morphology: the pure EP formed a porous, discontinuous carbon layer that failed to fully cover the underlying aluminum foil (Figure 6(a_1_)). By contrast, the EP-0.5 and EP-1 composites formed uniform and intact char layers with significantly reduced cracks and pores (Figure 6(c_1_,d_1_)). More remarkably, the side-view comparison showed a 3.75-fold increase in char height, from just 1.2 cm for pure EP (Figure 6(a_2_)) to 4.5 cm for EP-1 (Figure 6(d_2_)). This provided direct visual evidence of the remarkable char-forming capability of the flame-retardant P-Si. SEM analysis (Figure S2) provided crucial microstructural insights into this flame-retardant mechanism. The char layer of pure EP exhibited an extensively fractured and fragile morphology (Figure S2(a1,a2)), which facilitated oxygen penetration and flammable gas escape. In striking contrast, the flame-retardant P-Si-modified composites formed a remarkably coherent and dense char structure (Figure S2c–f). This layer functioned as an effective protective barrier, preventing further combustion while blocking the exchange of heat, oxygen, and generated gases.

The combined macro- and microscopic evidence demonstrated that the flame-retardant P-Si promoted the formation of an expanded char layer with optimal flame-retardant structural characteristics. This protective char operates through synergistic mechanisms: firstly, its increased thickness provides enhanced thermal insulation; secondly, its continuous and compact morphology effectively impeded the transfer of both heat and oxygen. These multifunctional attributes account for the dramatically improved fire performance observed in cone calorimeter tests.

**Figure 7:**
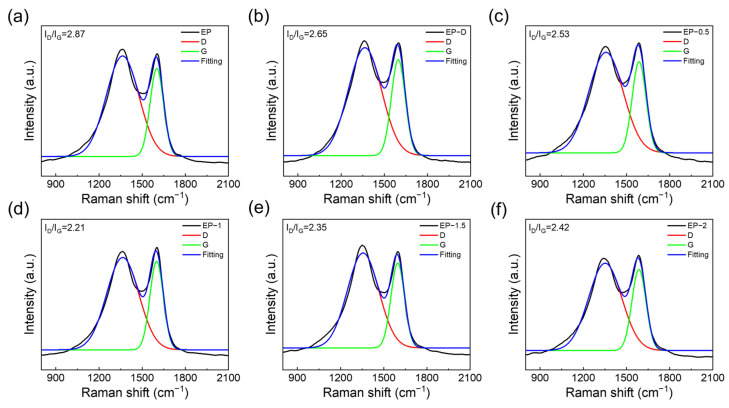
Raman spectra of the char residue of EP and EP/P–Si. Raman spectra of the char residue of (**a**) EP, (**b**) EP-D, (**c**) EP-0.5, (**d**) EP-1, (**e**) EP-1.5, (**f**) EP-2.

Figure 7 shows the Raman spectra of the coke residues after cone calorimeter testing for EP and EP/P–Si composite materials. The spectra exhibited two characteristic peaks: the D band at 1350 cm^−1^ represents defects in crystalline graphite, while the G band at 1580 cm^−1^ corresponds to the in-plane stretching vibration of sp^2^ hybridized carbon atoms. The ID/IG ratio represents the degree of graphitization of carbon. A lower ID/IG ratio indicates a higher degree of graphitization. The more graphitized the coke, the more stable it becomes. Stable coke was beneficial for forming a dense and continuous carbon layer, which could prevent further combustion of the matrix [50,51]. From Figure 7, it could be observed that the ID/IG values of the various samples were 2.87, 2.65, 2.53, 2.21, 2.35, and 2.42, respectively. Compared to pure epoxy, the coke residues of EP/P–Si composites showed a higher degree of graphitization. This improvement could be attributed to the presence of Si in the flame-retardant additive, which facilitates the formation of a more stable and ordered carbonaceous char during thermal decomposition. The Si component promotes carbonization reactions in the polymer matrix, thereby suppressing chain scission and volatile release. As a result, the EP/P–Si composites demonstrate superior flame retardancy, as evidenced by the formation of a more graphitized and thermally stable char layer that effectively insulated the underlying material and retarded further combustion.

**Figure 8:**
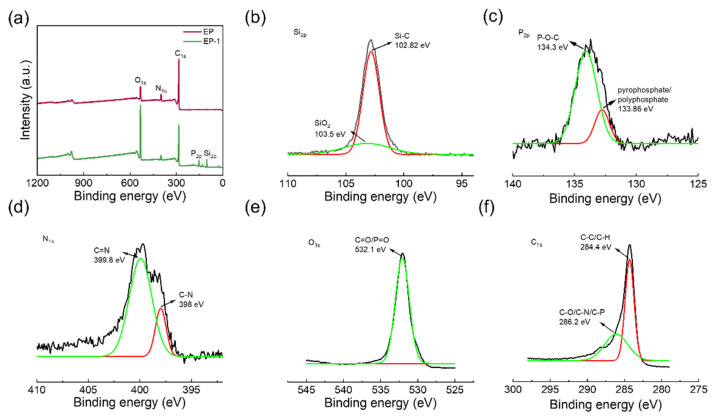
(**a**) XPS profiles of the char residue of EP and EP-1. (**b**) Si_2p_, (**c**) P_2p_, (**d**) N_1s_, (**e**) O_1s_, and (**f**) C_1s_ high-resolution XPS profiles of the char residue of EP-1.

The elemental compositions of EP and EP-1 were determined by using XPS (Figure 8). Both the EP and EP-1 contained oxygen, carbon, and nitrogen (Figure 8a), whereas the EP-1 spectrum showed the presence of silicon and phosphorus. Figure 8b–f show the high-resolution XPS spectra of the char residue of EP-1, including the Si_2_p, P_2_p, N_1_s, O_1_s, and C_1_s spectra. In the Si_2_p spectrum of EP-1 (Figure 8b), two peaks were observed at 102.82 eV and 103.5 eV, which could be attributed to Si-C bonds and SiO_2_, which were formed due to the pyrolysis of flame-retardant epoxy [51]. The P_2P_ spectrum of EP-1 (Figure 8c) showed a peak around 133.86 eV, which was attributed to pyrophosphate and/or polyphosphate, and may have promoted the carbonization of the EP matrix. In the high-resolution N_1_s spectrum (Figure 8d), EP-1 showed two peaks at 398 eV and 399.8 eV, which were attributed to C–N and C=N, respectively. The O_1_s spectrum of EP-1 also displayed a peak at 531.2 eV (Figure 8e), which was attributed to P=O/C=O [52,53]. In the C_1_s spectrum (Figure 8f), the peak at 284.2 eV in EP-1 was attributed to C=C or C-H, which result from the formation of graphite-like structures during combustion. The peak at 286.2 eV in EP-1 was attributed to C–O/C–P bonding. The above results show that flame-retardant P-Si was able to promote epoxy dehydration and coke formation in acidic phosphorus compounds (polyphosphates) during combustion and produced silicon dioxide (SiO_2_), which enhanced the stability of the carbon layer and various carbonaceous structures that contribute to the formation of protective graphitized carbon layers. This explained the significantly improved flame retardancy observed in EP-1 compared to pure EP.

**Figure 9:**
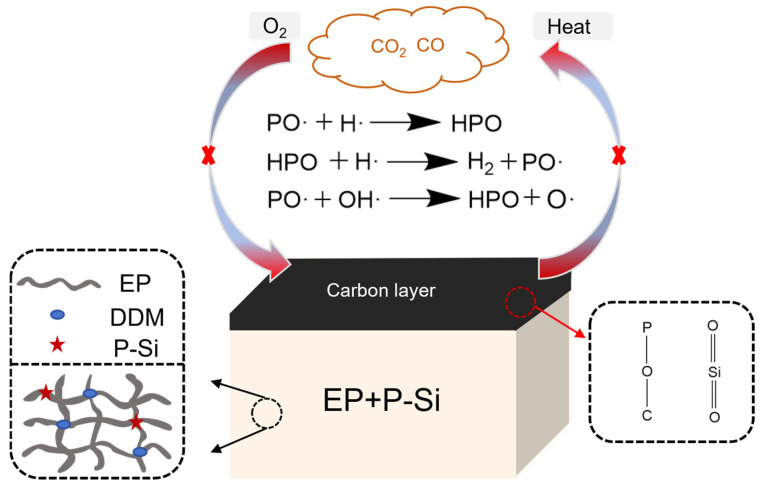
Flame-retardant mechanism of the EP/P–Si system.

Based on the comprehensive analysis above, the detailed flame-retardant mechanism of the P–Si flame retardant within the EP can be briefly summarized, as illustrated in Figure 9. In the gas phase, the thermal decomposition of the P–Si flame retardant generated incombustible gases, including CO_2_ and CO. These gases effectively diluted the concentration of oxygen and flammable volatile compounds in the surrounding environment. Additionally, the decomposition of P–Si released active free radicals (e.g., PO· and HPO·), which interacted with highly reactive combustion intermediates (e.g., H· and OH· radicals), quenching the free radical chain reaction and further inhibiting flame propagation [54,55]. These effects mitigated the combustion intensity of EP in the gas phase.

In the condensed phase, the degradation of P–Si yields thermally stable compounds, such as phosphorus-rich polyphosphates and SiO_2_. These decomposition products form a protective barrier on the material surface, shielding the underlying polymer from heat and preventing the escape of volatile fuel gases [40,56]. Furthermore, phosphorus and silicon synergistically promote char formation, enhancing the production of a continuous, compact, and thermally stable carbonaceous layer. This char layer acts as an effective insulator, minimizing heat transfer and oxygen diffusion and thereby significantly improving the flame retardancy of the EP composites. Through these combined mechanisms—gas-phase flame inhibition, condensed-phase barrier formation, and char reinforcement—the flame-retardant P–Si system provides robust fire resistance to the epoxy resin matrix.

**Figure 10:**
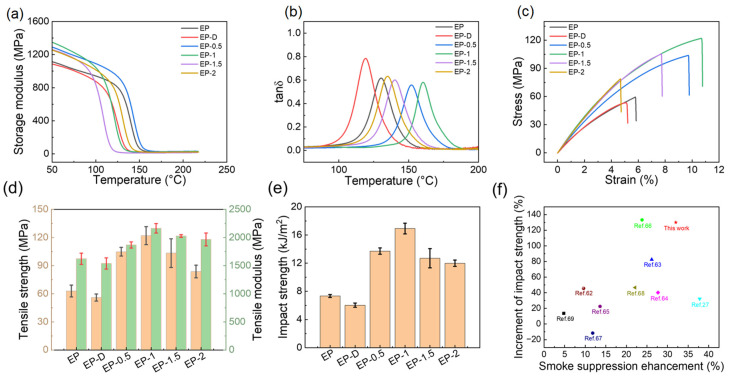
(**a**) Storage modulus, (**b**) tanδ, (**c**) stress–strain curve, (**d**) fracture stress and elastic modulus, and (**e**) impact strength. (**f**) Comparison of parameter obtained in this study with those reported in literature [27,59,60,61,62,63,64,65,66].

The dynamic mechanical behavior of EP/P–Si was studied using DMA. The *E* and loss tangent, as a function of temperature, are illustrated in Figure 10a,b, with the relevant data provided in Table S4. Compared with pure epoxy materials, the EP/P-Si composite materials exhibited a higher storage modulus (Figure 10a). This is because the flame retardant reacts with the epoxy resin to form a stable network, thereby enhancing the mechanical properties of the material. With the addition of the flame retardant, the glass transition temperature of the epoxy material first increased and then decreased (Figure 10b). This may be because the addition of the flame retardant increased the crosslinking density of the epoxy material, restricting the movement of the molecular chains, which resulted in an increase in the glass transition temperature. However, as the amount of flame retardant continued to increase, the compatibility between the flame retardant and the epoxy matrix worsened, leading to structural inhomogeneity within the material and a subsequent decrease in the glass transition temperature [41,57,58].

### 3.7. Mechanical Properties and Strengthening Mechanism

The crosslinking density (*V_e_*) of the cured EP is calculated using the flow equation derived from rubber elasticity theory [67,68]:

(9) $$V_{e}=\frac{E^{\prime }}{3RT}$$

where *E’* is the storage modulus at a temperature 50 °C above the glass transition temperature (*Tg*), R is the ideal gas constant (8.314 J/K·mol), and T is the thermodynamic temperature at *Tg* + 50 °C. As can be seen from Table S4, the crosslinking density of the cured EP matrix first increased and then decreased with the addition of increasing amounts of the flame-retardant P–Si. This result was consistent with the effect of the flame retardant on the storage modulus and *Tg*. A higher crosslinking density can enhance the rigidity of the material.

Figure 10c–e show that the tensile strength and toughness of the epoxy material with the flame-retardant P–Si were higher than those of the pure epoxy material. The tensile and impact strengths of EP-1 increased by 48.41% and 130%, respectively, compared to those of the pure epoxy. However, with continued addition of the flame retardant, the performance first increased and then decreased because the Si-O-Si bonds in the P-Si of the flame retardant helped to increase the material’s toughness. However, adding an excessive amount of flame retardant could reduce its compatibility with the epoxy matrix, leading to structural inhomogeneity.

The impact section of the specimen was examined by SEM, as shown in Figure S3. The fracture surface of neat EP exhibited a smooth morphology with linear crack propagation, showing no significant plastic deformation during fracture, which was characteristic of brittle failure. EP-D demonstrated reduced crack resistance with increased microcrack density and deteriorated mechanical strength. However, the fracture surface of the EP/P-Si composite displayed markedly enhanced wrinkling and wave-like patterns, effectively inhibiting rapid crack propagation. The toughening effect of P-Si can be attributed to its flexible Si-O-Si segments. These chain segments undergo substantial deformation under stress, enabling efficient energy absorption and dissipation and thereby improving the overall toughness of the composite material [20,69]. Compared to other reported flame retardants [27,59,60,61,62,63,64,65,66] (Figure 10f), a small amount of P-Si will endow materials with excellent toughness and smoke suppression.

### 3.8. Transparency Analysis

**Figure 11:**
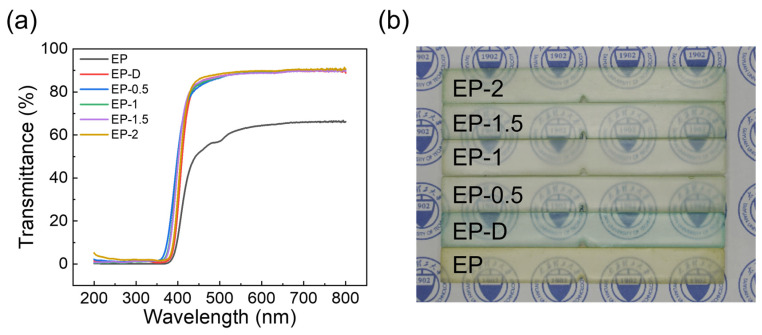
Epoxy composite optical property data: (**a**) transparency as a function of light wavelength; (**b**) sample appearance diagram.

Transparency is another important feature of EPs that is widely used in many fields. Therefore, the development of epoxy composites with excellent flame retardancy, mechanical properties, and transparency is crucial. The light transmittance of the epoxy sample was measured in the range of 200–800 nm using a UV-vis spectrophotometer. As shown in Figure 11a, in the visible-light region (400–800 nm), the light transmittance of the EP sample was approximately 90%. This high degree of transparency did not affect the appearance of the matrix. Figure 11b shows a digital photograph of the composite material. The samples of the EP and EP-D appear as yellow and green, respectively, but the epoxy material with the flame-retardant P–Si tends to be transparent. The school emblem logo indicates that the addition of P–Si improved the transparency.

## 4. Conclusions

This study demonstrates the successful simultaneous enhancement of both flame retardancy and mechanical performance in EP through the incorporation of the P-Si structure. Flame retardancy tests revealed that the EP composite achieved a UL-94 V-0 rating and an LOI of 33% with the addition of only 1 wt% P, exhibiting excellent smoke and heat suppression capabilities. The superior flame retardancy performance of the P-Si additive is attributed to the synergistic effect of phosphorus and silicon in the condensed phase, enhancing the generation of a continuous, dense, and thermally stable char layer, as well as the quenching of active free radicals and dilution by non-combustible gases in the gas phase. Concurrently, the EP-1 material exhibited a 48.41% increase in tensile strength and a 130% increase in impact strength, effectively addressing the trade-off between fire safety and mechanical robustness. These advancements highlight the potential of EP/P-Si composites for diverse applications.

## Figures and Tables

**Table 1:** EP/P–Si composites.

Sample	EP (g)	DOPO (g)	P–Si (g)	DDM (g)	P (wt%)
EP	30.00	/	/	7.59	/
EP-D	30.00	2.25	/	7.59	1.00
EP-0.5	30.00	/	1.50	7.59	0.50
EP-1	30.00	/	3.33	7.59	1.00
EP-1.5	30.00	/	5.00	7.59	1.50
EP-2	30.00	/	7.00	7.59	2.00

**Table 2:** Cure characteristics of EP/P–Si composites.

Sample	^a^ *T*_onset_ (°C)	^b^ *T*_P_ (°C)	^c^ *T*_end_ (°C)	Δ*T* (°C)	^d^ Δ*H ∞* (J/g)	ΔT *	ΔH *	*^e^*CI	^f^ Cure State
EP	137.50	160.68	180.99	43.49	294.56	/	/	/	/
EP-D	134.50	159.86	180.50	46.00	306.83	1.06	1.04	1.10	Good
EP-0.5	132.49	159.20	174.69	42.20	329.45	0.97	1.12	1.10	Excellent
EP-1	130.99	157.17	169.49	38.50	315.06	0.89	1.07	0.95	Excellent
EP-1.5	128.49	154.91	165.90	37.41	311.16	0.86	1.06	0.91	Excellent
EP-2	126.01	154.28	162.38	36.37	325.68	0.84	1.11	0.94	Excellent

^a^ *T*_onset_: the onset curing temperature. ^b^ *T*_P_: the exothermic peak temperature. ^c^ *T*_end_: the endset curing temperature. ^d^ Δ H ∞: total heat of reaction. ^e^ CI: cure index. ^f^ Cure state: CI < ΔT * (Poor), CI > Δ*H* * (Good), ΔT * < CI < ΔH * (Excellent).

**Table 3:** TG and DTG data of the epoxy composites.

Sample	^a^ T_5%_	^b^ T_Max_	^c^ R_Max_	^d^ CY700 (%)
EP	371.06	384.34	19.25	18.39
EP-D	345.42	369.67	13.99	19.73
EP-0.5	363.53	380.37	15.83	20.02
EP-1	359.84	378.33	13.89	21.58
EP-1.5	357.33	378.01	14.11	21.32
EP-2	349.85	374.67	11.28	20.87

^a^ T_5%_: the temperature of 5 wt% mass loss. ^b^ T_Max_: the temperature of maximum mass loss. ^c^ R_Max_: R_Max_ is the maximum mass loss rate. ^d^ CY700: the residual char yield of the sample at 700 °C.

**Table 4:** Combustion testing data of the epoxy composites.

Sample	LOI (%)	^a^ t_1_ (s)	^b^ t_2_ (s)	Dripping or Not	UL-94 Rating
EP	23	None	None	Yes	**^c^** NR
EP-D	31	6	5	No	V-1
EP-0.5	29	8.7	6	No	V-1
EP-1	33	1	1	No	V-0
EP-1.5	31	1	3.2	No	V-0
EP-2	30	3	4.4	No	V-0

^a^ t_1_: the first flame combustion times. ^b^ t_2_: the second flame combustion times. ^c^ NR: NR indicates no flame retardancy rating.

**Table 5:** Determination of combustion parameters of the epoxy composites by cone calorimetry.

Sample	^a^ TTI (s)	^b^ pHRR (kW/m^2^)	^c^ THR (MJ/m^2^)	^d^ av-COY (kg/kg)	^e^ av-CO_2_Y (kg/kg)	^f^ av-EHC (MJ/kg)	^g^ TML (wt%)
EP	82 ± 1	1322.55 ± 125.61	116.20 ± 3.17	0.520 ± 0.005	3.24 ± 0.17	27.5 ± 0.24	94.2 ± 1.1
EP-D	84 ± 2	1272.72 ± 93.43	103.11 ± 1.94	0.408 ± 0.004	3.16 ± 0.13	24.2 ±0.83	88.7 ± 0.3
EP-0.5	87 ± 1	1032.60 ± 46.74	96.73 ± 3.34	0.314 ± 0.006	3.13 ± 0.09	23.3 ± 0.71	85.4 ± 0.3
EP-1	93 ± 1	629.11 ± 28.03	74.72 ± 2.81	0.258 ± 0.003	2.33 ± 0.15	17.0 ± 0.32	80.1 ± 0.5
EP-1.5	91 ± 1	697.45 ± 37.23	83.22 ± 3.49	0.320 ± 0.002	2.71 ± 0.24	20.7 ± 0.48	82.3 ±1.3
EP-2	88 ± 2	827.23 ± 41.84	92.92 ± 1.21	0.327 ± 0.005	2.97 ± 0.31	21.7 ± 0.65	83.1 ±0.8

^a^ TTI: time to ignition. ^b^ pHRR: peak of heat release rate. ^c^ THR: total heat release. ^d^ av-COY: average yield of CO. ^e^ av-CO_2_Y: average yield of CO_2_. ^f^ av-EHC: average value of effective heat of combustion. ^g^ TML: total mass loss.

## Data Availability

The original contributions presented in this study are included in the article/Supplementary Material. Further inquiries can be directed to the corresponding author.

## Supplementary Materials

The following supporting information can be downloaded at: https://www.mdpi.com/article/10.3390/ma18122753/s1. Table S1: non-isothermal curing kinetic parameters; Table S2: comparison of the flame retardancy between this work and relevant literature in the past five years; Table S3: three different effects; Table S4: storage modulus, glass transition temperature, and crosslinking density of the epoxy flame retardant materials; Figure S1: the UL-94 testing screenshot photos from video of EP and EP/P-Si materials; Figure S2: SEM images of the char residue. (a) EP, (b) EP-D, (c) EP-0.5, (d) EP-1, (e) EP-1.5, (f) EP-2; Figure S3: impact fracture surface of (a) EP, (b) EP-D, (c) EP-0.5, (d) EP-1, (e) EP-1.5, (f) EP-2. Refs. [70,71,72,73,74,75,76,77,78,79] have been cited in Supplementary Materials File.
