# Supplementary material for: Phosphorus–Silicon Additive Increases the Mechanical and Fire Resistance of Epoxy Resins
Source: Materials (Basel). 2025 Jun 12;18(12):2753. doi: 10.3390/ma18122753 (PMC12195051; doi:10.3390/ma18122753)
Supplement: Supplementary file 1 [file materials-18-02753-s001.zip › materials-3624354-supplementary.pdf]

Supplementary materials

Phosphorus-silicon Additive Increases Mechanical and Fire Resistance of Epoxy Resins

Zhe Wang [1], Shuaijun Guo [1], Wenwen Yu [1] and Xiaohong Liang [1,*]

[1] College of Materials Science & Engineering, Taiyuan University of Technology, Taiyuan 030024, China; wangzhe0243@link.tyut.edu.cn (Z.W.); 2023310047@linlk.tyut.edu.cn (S.J.G.); yuwenwen@tyut.edu.cn. (W.W.Y.)

* Correspondence: xhliang1983@163.com

Table S1. Non-isothermal curing kinetic parameters.

Sample	E_a (kJ/mol)		R^2	
	Kissinger	Ozawa	Kissinger	Ozawa
EP	42.40	52.56	0.9994	0.9918
EP-D	41.35	50.38	0.9995	0.9937
EP-1	39.66	49.31	0.9996	0.9929

Table S2. Comparison of the flame retardancy between this work and relative literature in the past five years

Sample	FR content (%)	LOI (%)	UL-94 Rating	Refence
Pure EP	/	23.5	NR	[1]
7.5% BADO/EP	7.5	30.7	V-0	[2]
EP/7% RP@CH/LS	7	30.6	V-0	[3]
Ep/10% CCD	10	31.6	V-0	[4]
50% VCP/EP	17	27.1	V-1	[5]
D8B2	20	27.5	V-0	[6]
EP/PCS-4	20	31.2	V-0	[7]
EP3	18	30.6	V-0	[8]
EP/PBEA-9.0	9	29	V-0	[9]
EP-6	6	29.8	V-0	[10]
ETP-6	6	29.6	V-0	[11]
PDEDP-5	5	2.91	V-0	[12]
EP/DDM/DPATS-4	4	31.1	V-0	[13]
EP-1	10	33	V-0	This work

Table S3. Three different effects.

Sample	Flame inhibition effect	Charring effect	Barrier and protective effect
EP	/	/	/
EP-D	0.12 ± 0.022	0.852 ± 0.006	0.064 ± 0.014
EP-0.5	0.15± 0.018	0.876 ± 0.003	0.085 ± 0.015
EP-1	0.31 ± 0.006	0.944 ± 0.002	0.265 ± 0.027
EP-1.5	0.24 ± 0.001	0.901 ± 0.003	0.268 ± 0.027
EP-2	0.21 ± 0.002	0.884 ± 0.005	0.223 ± 0.082

Table S4. Storage modulus, glass transition temperature, and crosslinking density of the epoxy flame retardant materials.

Sample	E at 50 °C (MPa)	E' at T_g + 50 °C (MPa)	T_g (°C)	V_e (mol/m^3)
EP	1123.53 ± 26.35	17.18 ± 0.26	128.48 ± 1.85	1714.95 ± 18.06
EP-D	1036.36 ± 57.28	16.37 ± 1.62	115.37 ± 0.87	1689.05 ± 163.39
EP-0.5	1311.20 ± 43.56	24.29 ± 1.84	149.68 ± 2.48	2302.56 ± 160.97
EP-1	1326.81 ± 28.04	26.83 ± 1.24	157.26 ± 1.34	2499.01 ± 107.73
EP-1.5	1248.44 ± 60.16	21.56 ± 0.85	143.71 ± 0.98	2073.49 ± 76.88
EP-2	1198.13 ± 15.82	19.52 ± 0.86	132.94 ± 2.15	1926.93 ± 74.71

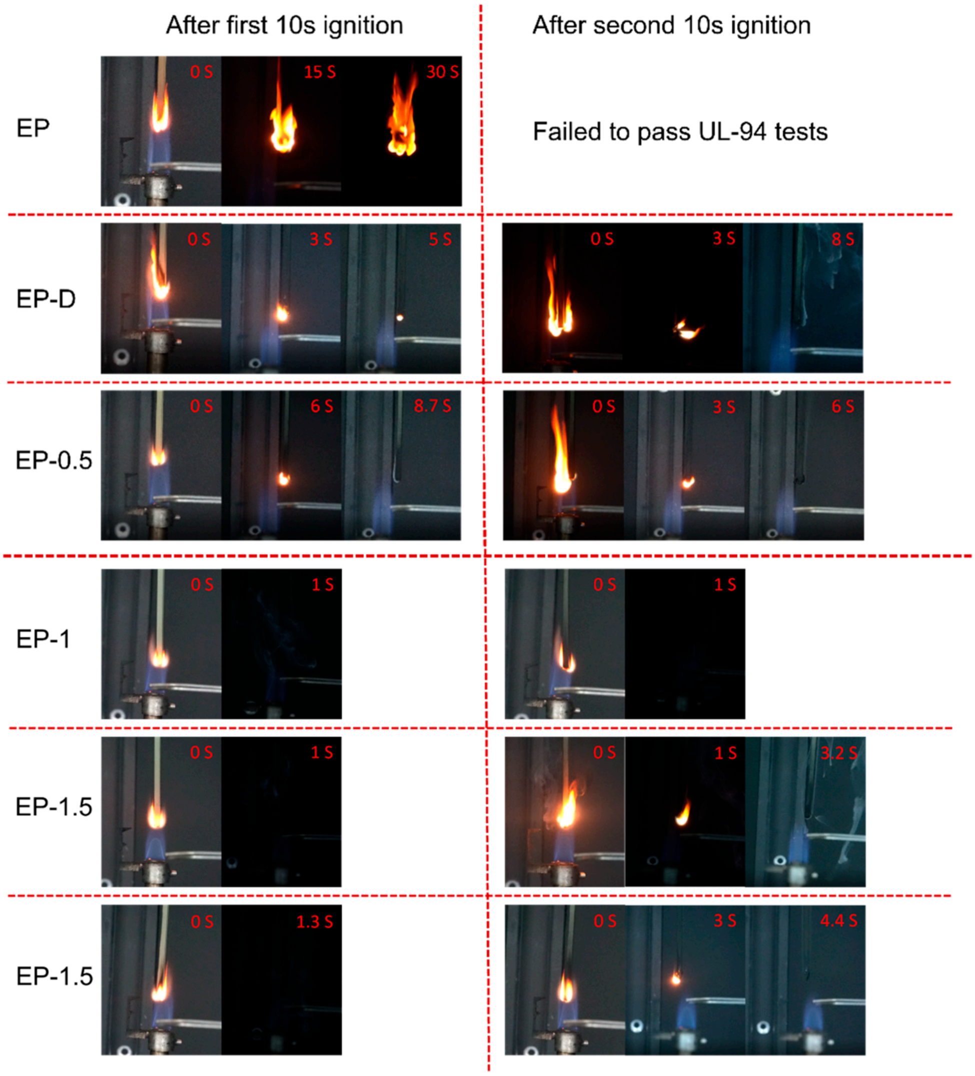

Supplementary Figure S1. The UL-94 testing screenshot photos from video of EP and EP/P-Si materials.

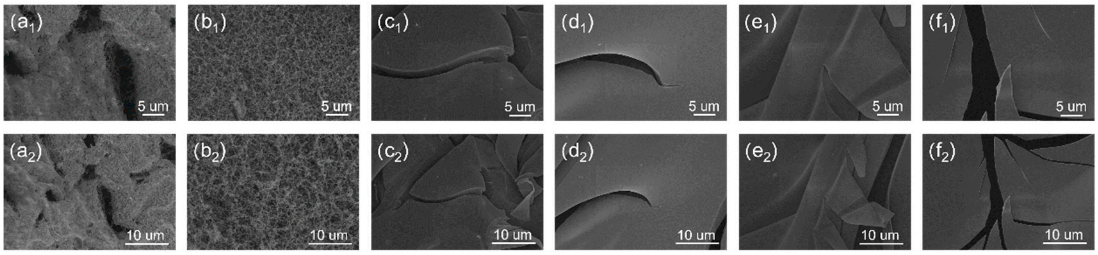

Supplementary Figure S2. SEM images of the char residue. (a) EP, (b) EP-D, (c) EP-0.5, (d) EP-1, (e) EP-1.5, (f) EP-2.

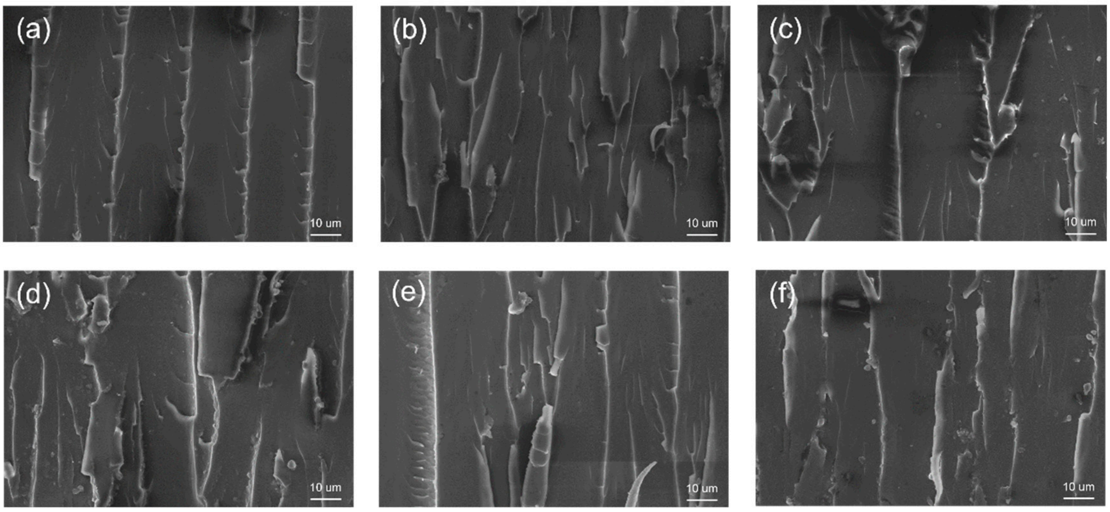

Supplementary Figure S3. Impact fracture surface of (a) EP, (b) EP-D, (c) EP-0.5, (d) EP-1, (e) EP-1.5, (f) EP-2.

References

1. Zhi, M.-Y.; Yang, X.; Fan, R.; Yue, S.; Zheng, L.-L.; Liu, Q.-Y. A comprehensive review of reactive flame-retardant epoxy resin: fundamentals, recent developments, and perspectives. *Polym. Degrad. Stab.* **2022**, *201*, 109976. https://doi.org/10.1016/j.polymdegradstab.2022.109976.
2. Liu, D.-Y.; Jiang, F.; Zhang, T.-L.; Yu, C.-X.; Hu, Z.-Y.; Zhang, L.; Shi, S.-Y. A DOPO-based reactive flame retardant containing benzimidazole groups: synthesis and its flame-retardation on epoxy resin. *J. Appl. Polym. Sci.* **2024**, *141*, e54890. https://doi.org/10.1002/app.54890.
3. Cheng, C.; Yan, J.; Lu, Y.-L.; Ma, W.-N.; Du, S.-G. Effect of chitosan/lignosulfonate microencapsulated red phosphorus on fire performance of epoxy resin. *Thermochim. Acta* **2021**, *700*, 178931. https://doi.org/10.1016/j.tca.2021.178931.
4. Chen, R.; Luo, Z.-J.; Yu, X.-J.; Tang, H.; Zhou, Y.; Zhou, H. Synthesis of chitosan-based flame retardant and its fire resistance in epoxy resin. *Carbohydr. Polym.* **2020**, *245*, 116530. https://doi.org/10.1016/j.carbpol.2020.116530.
5. Cheng, H.-X.; Guo, J.-H.; Ye, Y.; Zhao, T.-J. Molecular design and properties of intrinsic flame-retardant P-N synergistic epoxy resin. *J. Appl. Polym. Sci.* **2024**, *141*(5), e54885. https://doi.org/10.1002/app.54885.
6. Zhou, W.; Lv, X.-D.; Ding, H.; Xu, P.-W.; Zhang, C.-J.; Ren, Y.-Z.; Yang, W.-J. Synthesis of eugenol-based phosphorus-containing epoxy for enhancing the flame-retardancy and mechanical performance of DGEBA epoxy resin. *React. Funct. Polym.* **2022**, *180*, 105383. https://doi.org/10.1016/j.reactfunctpolym.2022.105383.
7. Wang, H.; Wang, Y.-J.; Su, Y.; Yu, C.; Han, J.; Liu, J.-P. Preparation of a polymeric phosphoramide flame-retardant and its effect on the flame-retardant properties of epoxy Resin. *Polymers* **2024**, *16*(9), 1224. https://doi.org/10.3390/polym16091224.
8. Yu, H.-H.; Xu, X.-H.; Xia, Y.-F.; Pan, M.-Z.; Zarshad, N.; Pang, B. Synthesis of a novel modified chitosan as an intumescent flame retardant for epoxy resin. *e-Polymers* **2020**, *20*(1), 303-316. https://doi.org/10.1515/epoly-2020-0036.
9. Yang, G.; Wu, W.-H.; Wang, Y.-H.; Jiao, Y.-H.; Lu, L.-Y.; Qu, H.-Q. Synthesis of a novel phosphazene-based flame retardant with active amine groups and its application in Reducing the fire Hazard of epoxy resin. *J. Hazard. Mater.* **2019**, *366*, 78-87. https://doi.org/10.1016/j.jhazmat.2018.11.093.
10. Zhang, Y.-B.; Yan, H.-X.; Feng, G.-P.; Liu R.; Yang, K.-M.; Feng, W.-X. Non-aromatic Si, P, N-containing hyperbranched flame retardant on reducing fire hazards of epoxy resin with desirable mechanical properties and lower curing temperature. *Compos. Part B Eng.* **2021**, *222*, 109043. https://doi.org/10.1016/j.compositesb.2021.109043.
11. Yu M.; Zhang, T.-T.; Li, J.; Tan, J.-H.; Zhu, X.-B. Enhancing toughness, flame retardant, hydrophobic and dielectric properties of epoxy resin by incorporating multifunctional additive containing phosphorus/silicon. *Mater. Des.* **2023**, *225*, 111529. https://doi.org/10.1016/j.matdes.2022.111529.
12. Yu M.; Zhang, T.-T.; Li, J.; Tan, J.-H.; Zhu, X.-B. Synthesis of a multifunctional Phosphorus/Silicon flame retardant via an industrial feasible technology. ACS *Sustain. Chem. Eng.* **2023**, *11*(32), 11965-11977. https://doi.org/10.1021/acssuschemeng.3c02140.
13. Cao, Y.-F.; Sun, L.; Ding, L.; Yu, J.-K. A flexible silicon-oxygen chain segment-containing phosphonamidite toward modification of epoxy resin: flame retardancy, toughness and

transparency. *Eur. Polym. J.* **2025**, *223*, 113636. https://doi.org/10.1016/j.eurpolymj.2024.113636.
